# Ultra-Highly Sensitive Ammonia Detection Based on Light-Induced Thermoelastic Spectroscopy

**DOI:** 10.3390/s21134548

**Published:** 2021-07-02

**Authors:** Yao Mi, Yufei Ma

**Affiliations:** National Key Laboratory of Science and Technology on Tunable Laser, Harbin Institute of Technology, Harbin 150001, China; 1182100107@stu.hit.edu.cn

**Keywords:** quartz tuning fork (QTF), light-induced thermoelastic spectroscopy (LITES), ammonia (NH_3_), trace gas detection

## Abstract

This invited paper demonstrated an ultra-highly sensitive ammonia (NH_3_) sensor based on the light-induced thermoelastic spectroscopy (LITES) technique for the first time. A quartz tuning fork (QTF) with a resonance frequency of 32.768 kHz was employed as a detector. A fiber-coupled, continuous wave (CW), distributed feedback (DFB) diode laser emitting at 1530.33 nm was chosen as the excitation source. Wavelength modulation spectroscopy (WMS) and second-harmonic (2*f*) detection techniques were applied to reduce the background noise. In a one scan period, a 2*f* signal of the two absorption lines located at 6534.6 cm^−1^ and 6533.4 cm^−1^ were acquired simultaneously. The 2*f* signal amplitude at the two absorption lines was proved to be proportional to the concentration, respectively, by changing the concentration of NH_3_ in the analyte. The calculated R-square values of the linear fit are equal to ~0.99. The wavelength modulation depth was optimized to be 13.38 mA, and a minimum detection limit (MDL) of ~5.85 ppm was achieved for the reported NH_3_ sensor.

## 1. Introduction

Ammonia (NH_3_), a component gas of the atmosphere, has been widely used in various important fields, such as medicine production, chemical industries, and so on [1]. When it comes to a medical diagnostic, NH_3_ can be used as a biomarker to indicate kidney and liver diseases [2,3]. However, on the other hand, ammonia also has lots of hazards. For example, NH_3_ poses a serious threat to human health, which can burn skin, eyes, and the respiratory mucosa. If people inhale too much, it can cause lung swelling and even death [4]. NH_3_ is also the main cause of air pollution. Hence, carrying out ammonia detection is necessary for both industrial production and the environment. Nevertheless, the typical concentration of NH_3_ is at low levels of parts per million (ppm) or parts per billion (ppb). Therefore, NH_3_ sensors should be ultra-highly sensitive to satisfy these applications.

Various sensors have been used for NH_3_ detection. Chemical sensors are widely applied in gas detection, which has the advantages of low cost and small size [5]. However, when chemical sensors are employed to detect NH_3_, it may be influenced by other gasses, such as oxygen [6]. Laser absorption spectroscopy (LAS) is an effective method with the advantages of being non-invasive, highly sensitive, having a fast response, and selective detection, and has been widely used for trace gas sensing. Tunable diode laser absorption spectroscopy (TDLAS) is a serviceable LAS-based technique to detect the concentration of NH_3_ [7]. To obtain an excellent detection performance, a multi-pass gas cell (MPGC) is employed in the TDLAS technique [8]. When the laser beam travels through the MPGC, the effective optical path length can be extended to a significant scale, which can bring the detection limit down to ppm levels [9,10]. However, adopting an MPGC means the total equipment is costly and bulky by virtue of the large size of an MPGC and the large quantity of optical elements used to align the laser beam.

Quartz-enhanced photoacoustic spectroscopy (QEPAS), which was reported in 2002 for the first time [11], is another effective method for trace gas detection. A quartz tuning fork (QTF) is employed in QEPAS to transform acoustic wave signals to piezoelectric signals, whose amplitude reflects the gas concentration. The QTF has the advantages of being commercially available, low cost, having a tiny volume, a dipole structure, a high Q-factor, a wide dynamic range, and a narrow resonance frequency band. Due to these advantages, QEPAS could be excellent for sensitive detection and obtain great immunity to environmental noise [12,13,14,15,16,17,18]. A sensitive NH_3_-QEPAS sensor achieving the minimum detection limit (MDL) of ~418.4 ppb was reported in 2017 [19]. In QEPAS, the QTF needs to be placed in the gas cell and immersed in the target gas. When using QEPAS to detect the concentration of corrosive gases such as NH_3_, the QTF can be corroded, which may finally bring sensor failure.

Light-induced thermoelastic spectroscopy (LITES), an effective technique first reported in 2018 [20], has been widely employed for trace gas detection. This technique is also named quartz-enhanced photothermal spectroscopy (QEPTS). In LITES, the laser beam is focused on the QTF after traveling through the sample gas cell and being absorbed. Hence, when the laser arrives at the QTF, its power is converted into thermal energy in the quartz crystal [21,22,23]. Due to thermoelastic deformation, the periodic change of laser energy contributes to the periodic mechanical motion of QTF prongs, which would be enhanced by the resonance property of QTF [24,25]. Because of the piezoelectric effect, the QTF transforms mechanical vibrations into electrical signals. By demodulating the electrical signal, the concentration of sample gas can be obtained [26,27]. Compared to QEPAS, LITES has the same advantages as QEPAS while avoiding the QTF damage caused by target gas corrosiveness [28]. Therefore, LITES has been widely used in corrosive gas detection [29]. However, till now, NH_3_ detection using the LITES technique has not been reported.

In this invited manuscript, an ultra-highly sensitive NH_3_ sensor based on the LITES technique is demonstrated for the first time. By means of wavelength modulation spectroscopy (WMS) and second-harmonic detection (2*f*) techniques, the background noise of the sensor was able to dropdown. One current scan period covered two different absorption lines of NH_3_. By changing the concentration of NH_3_ in the analyte, a linear relationship between 2*f* signal amplitude and concentration was demonstrated. After optimizing the response time and modulation depth, an MDL of ~5.85 ppm was achieved for this reported NH_3_-LITES sensor.

## 2. Experimental Setup

### 2.1. Absorption Line Selection

**Figure 1:**
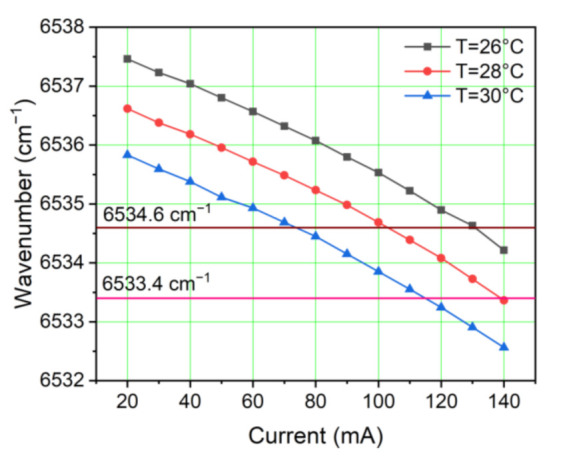
Emission characteristic of the 1530.33 nm CW-DFB diode laser.

Diode lasers have many merits, such as a wide tunable range from near-ultraviolet to near-infrared, small size, narrow linewidth, and high optical efficiency, making them have important applications in single-chip laboratory, medical diagnosis, dermatology, and gas sensing. In this experiment, a fiber-coupled, near-infrared, continuous wave (CW), distributed feedback (DFB) diode laser emitting at 1530.33 nm was chosen to be the excitation source. By changing the injection current at different temperatures, the emission characteristic of this diode laser was measured. The results are shown in Figure 1. Considering that the LITES signal has wide dynamic responses to laser power [20], the output power of the CW-DFB diode laser can meet the demand.

**Figure 2:**
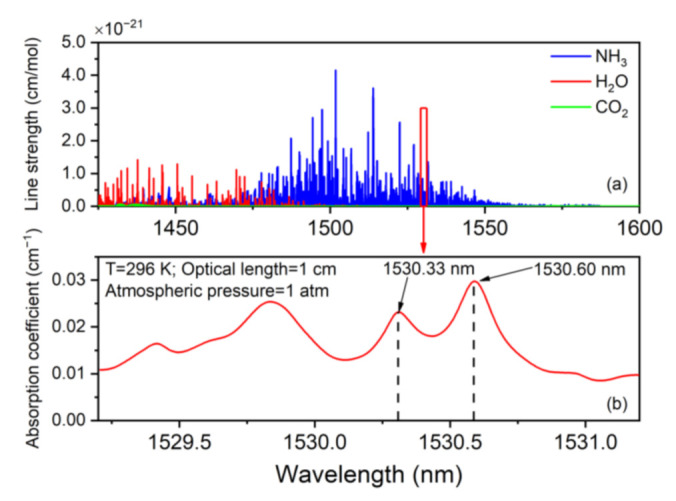
Simulation absorption spectra based on HITRAN database: (**a**) absorption line strength of different gases; (**b**) absorption coefficient of NH_3_ at 296 K, standard atmospheric pressure, and an optical path length of 1 cm for 10,000 ppm NH_3_:N_2_.

Considering that different gases may influence the detection, the absorption lines of NH_3_, H_2_O, and CO_2_ located between 1425 nm and 1600 nm were calculated respectively based on the HITRAN 2016 database [30]. As is shown in Figure 2a, the existence of CO_2_ or H_2_O could not influence the NH_3_ detection. Considering that the CW-DFB diode laser chosen in this experiment is able to cover the wavelength from 1530.69 nm (6533 cm^−1^) to 1529.99 nm (6536 cm^−1^), two absorption lines of NH_3_, which are respectively located at 1530.33 nm (6534.6 cm^−1^) and 1530.60 nm (6533.4 cm^−1^), were chosen in this investigation. The selected absorption lines are depicted in Figure 2b.

### 2.2. The Configuration of Experimental Setup

**Figure 3:**
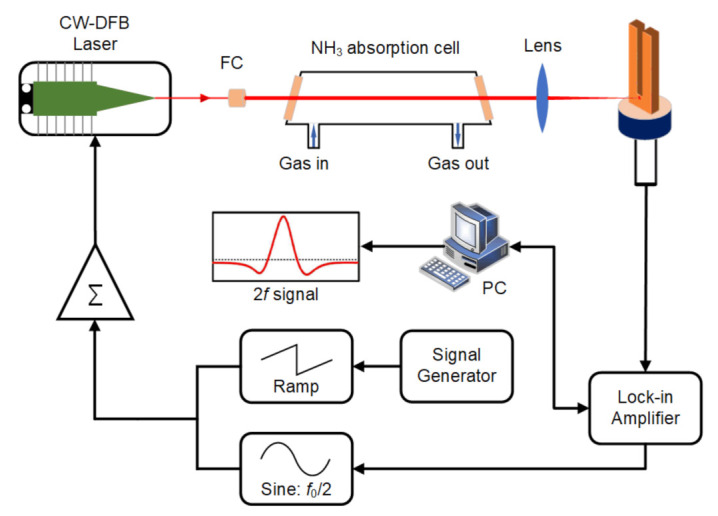
Schematic diagram of the LITES sensor system.

A schematic diagram of the LITES sensor system is exhibited in Figure 3. A fiber collimator (FC) was employed in order to collimate the laser beam generated by the fiber-coupled, CW-DFB diode laser. Afterward, the laser beam traveled through the absorption cell with a length of 20 cm and filled with target gas. To avoid optical interference, two wedged CaF_2_ windows were installed on both sides of the absorption cell. Subsequent to propagating through the cell, the laser beam is focused by a lens with a focal length of 40 mm on a QTF with a low intrinsic resonance frequency *f*_0_ of 32.768 kHz in a vacuum. For the purpose of acquiring the maximum signal, the position where the laser beam focuses on the QTF’s surface is supposed to be optimized. As was reported in [31], the optimum laser focusing position is the bare surface area on the base of QTF’s prongs. WMS and 2*f* detection techniques were adopted in this LITES sensor system. An adder was employed for the CW-DFB laser, which added a low-frequency ramp wave generated by a signal generator and a high-frequency sinusoidal wave (*f* = *f*_0_/2 = 15.36 kHz) generated by a lock-in amplifier together. The ramp wave contributed to continuously changing the emission wavelength of the CW-DFB diode laser across the absorption lines of NH_3_. The demodulated 2*f* component of the LITES signal could be acquired by a lock-in amplifier. In this reported NH_3_-LITES sensor system, the integration time of the lock-in amplifier was 200 ms. The experiment was accomplished at room temperature and atmospheric pressure. The LITES technique has wide dynamic responses to gas concentration [20]; therefore, a certified gas mixture of 10,000 ppm NH_3_:N_2_ was utilized as the analyte. The experimental results were verified by repeated measurements.

## 3. Experimental Results and Discussion

**Figure 4:**
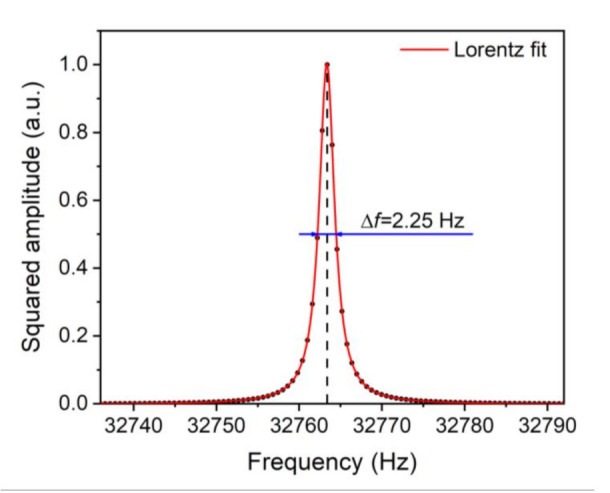
Normalized and squared amplitude as a function of frequency.

Firstly, the QTF’s properties were investigated. There are two methods to investigate the QTF’s properties. The first one applies a laser beam as the excitation source [27]; the other one applies electric excitation [32]. The first method was adopted in this experiment. As is shown in Figure 4, the intrinsic resonance frequency *f*_0_ and bandwidth Δ*f* were measured as 32.763 kHz and 2.25 Hz, respectively. The quality factor *Q* = *f*_0_/Δ*f* was calculated as 14,541, indicating its good performance.

**Figure 5:**
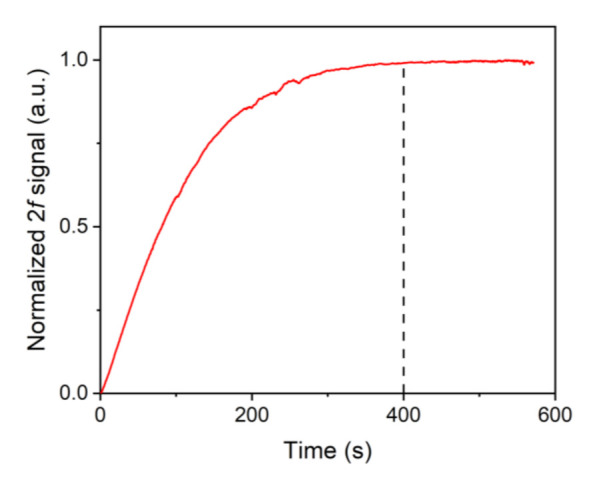
The correlation between normalized 2*f* signal value and ventilation time.

Considering that NH_3_ is able to be adsorbed onto the inner surface of the absorption cell, the detected 2*f* signal is unstable before NH_3_ is in a saturation adsorption state. Hence, it is crucial to investigate the relationship between the 2*f* signal value and ventilation time. The measured results are exhibited in Figure 5. The results indicate that the normalized signal value is essentially stable after 400 s. Hence, to acquire stable experimental data, the experiment is expected to be carried out at least 400 s after the NH_3_ injection.

**Figure 6:**
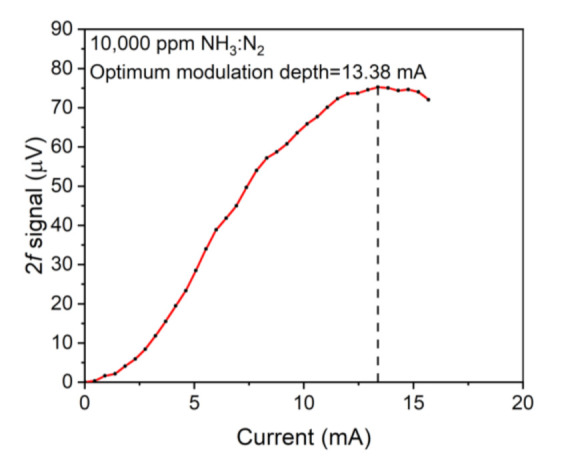
The correlation between wavelength modulation depth and 2*f* signal value.

Figure 6 reflects the correlation between the NH_3_-LITES signal value and wavelength modulation depth. In this paper, wavelength modulation depth is described by injection current. It could be seen that the NH_3_-LITES signal amplitude rose to a maximum first and then fell down with the increase in injection current. When the wavelength modulation depth was 13.38 mA, the 2*f* signal achieved the maximum value. Therefore, the optimum modulation depth of 13.38 mA was used in the following investigations.

**Figure 7:**
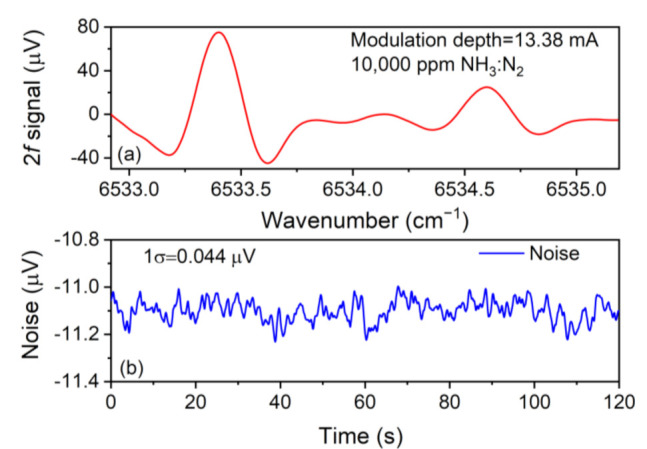
(**a**) 2*f* signal of 10,000 ppm NH_3_:N_2_; (**b**) noise level of NH_3_-LITES sensor.

When the modulation depth was 13.38 mA, the 2*f* signal was measured and exhibited in Figure 7. The 2*f* signal had two peaks corresponding to the two absorption lines located at 6534.6 cm^−1^ (1530.33 nm) and 6533.4 cm^−1^ (1530.60 nm), respectively. The peak value at 6533.4 cm^−1^ was 75.23 μV, while the other peak value was 24.89 μV. Obviously, the peak value at 6533.4 cm^−1^ was much bigger than what was located at 6534.6 cm^−1^, which agreed with the absorption line strength data from the HITRAN database well. Hence, the absorption line located at 6533.4 cm^−1^ was selected to carry out further investigation. The background noise level was determined by continually monitoring the amplitude for 120 s when the absorption cell was filled with nitrogen (N_2_). The results are shown in Figure 7b. The calculated 1σ noise value was 0.044 μV. In terms of the data depicted in Figure 7, a signal-to-noise ratio (SNR) value of ~1709 was calculated. In view of the definition that minimum detection limit (MDL) = analyte concentration/SNR, an MDL of ~5.85 ppm was acquired for this NH_3_-LITES sensor.

**Figure 8:**
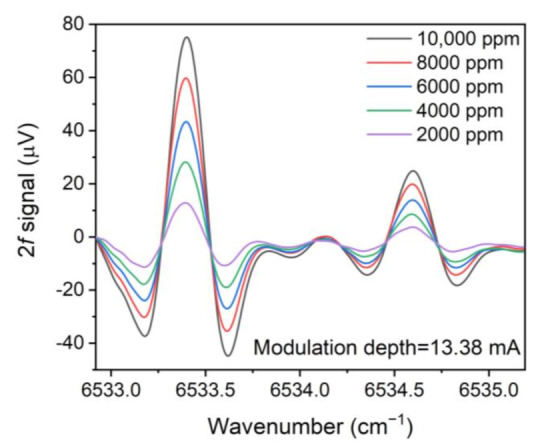
2*f* signal of mixture gas with different NH_3_ concentrations.

**Figure 9:**
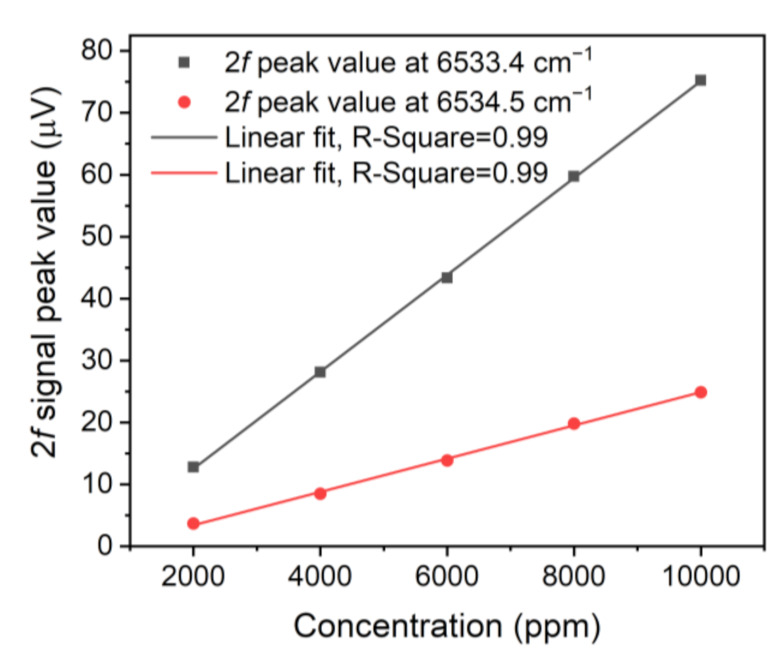
Linear fits of 2*f* signal peak values and NH_3_ concentration.

To verify the linear response of the LITES signal on the NH_3_ concentration, the 2*f* signals for different NH_3_ concentrations are shown in Figure 8. To obtain a mixture gas of different concentrations, two mass flow controllers were employed to control the gas flow rate of 10,000 ppm NH_3_:N_2_ and pure N_2_, respectively. Linear fits of LITES signal amplitude and NH_3_ concentration are shown in Figure 9, respectively, for the two absorption lines. The calculated R-square values are equal to ~0.99, indicating that the LITES signal performs a splendid linear response of NH_3_ concentration levels for the two selected lines.

## 4. Conclusions

This paper demonstrated an ultra-highly sensitive NH_3_ sensor based on the LITES technique for the first time. A fiber-coupled, near-infrared, CW-DFB diode laser emitting at 1530.33 nm was chosen to be the excitation source. A QTF with an intrinsic resonance frequency *f*_0_ of 32.768 kHz was used as a detector to transduce mechanical vibrations into electrical signals. By means of WMS and 2*f* detection techniques, the background noise of the sensor was reduced to a low level. Two different absorption lines of NH_3_ located at 6534.6 cm^−1^ and 6533.4 cm^−1^ were chosen and investigated. The response of the LITES signal on the NH_3_ concentrations was investigated, which indicated an excellent linear response. The wavelength modulation depth was optimized to be 13.38 mA, and finally, an MDL of ~5.85 ppm was achieved for this NH_3_-LITES sensor. This ppm-level NH_3_-LITES sensor has the potential to be applied in environmental monitoring, medical diagnostic, and other fields.

## Data Availability

The data presented in this study are available on request from the corresponding author.
